# Bioactive Content and Antioxidant Properties of Spray-Dried Microencapsulates of *Peumus boldus* M. Leaf Extracts

**DOI:** 10.3390/antiox13121568

**Published:** 2024-12-20

**Authors:** Valentina Polanco, Débora Cerdá-Bernad, Issis Quispe-Fuentes, Claudia Bernal, Jéssica López

**Affiliations:** 1 Escuela de Alimentos, Pontificia Universidad Católica de Valparaíso, Waddington 716, Playa Ancha, Valparaíso 2360100, Chile; valentina.polanco.o@mail.pucv.cl; 2 Agro-Food Technology Department, Centro de Investigación e Innovación Agroalimentaria y Agroambiental, Miguel Hernández University CIAGRO-UMH, 03312 Orihuela, Spain; dcerda@umh.es; 3 Food Engineering Department, Universidad de La Serena, Raúl Bitran 1305, La Serena 1700000, Chile; iquispe@userena.cl; 4 Laboratorio de Catálisis y Biocatálisis, Departamento de Química, Facultad de Ciencias, Universidad de La Serena, Casilla 599, Benavente 980, La Serena 1720236, Chile; cbernal@userena.cl

**Keywords:** *Peumus boldus* M., boldo, phenolic compounds, encapsulation, SEM, FTIR, maltodextrin, infusion extraction, spray-drying, antioxidant activity

## Abstract

Boldo (*Peumus boldus* M.), an plant endemic to central and southern Chile, has been recognized as a medicinal herb, especially its leaves that are rich in bioactive compounds with beneficial properties, such as antioxidant, anti-inflammatory, sedative, and antimicrobial properties, among others. This research aimed to evaluate solid-liquid extraction using a response surface methodology to obtain phenolic-rich extracts from boldo leaves and to encapsulate them through spray-drying. A Box-Behnken design was applied to optimize extraction process variables (temperature, time, and solid-liquid ratio). Extracts were characterized in terms of their total phenolic content, with the maximum value obtained being 37.78 mg GAE/g using extraction conditions of a temperature of 100 °C, a time of 60 min, and a solid-liquid ratio of 1:100. The developed microcapsules containing the optimal boldo extracts were characterized (moisture, water activity, scanning electron microscopy, zeta potential, FTIR, total phenolic compounds, antioxidant capacity, and phenolic profile by HPLC-DAD), highlighting their high phenolic content (5.38–5.49 mg GAE/g) and antioxidant capacity, as well as their bioactive content in terms of catechin (445 ± 37 mg/100 g), pyrogallol (304 ± 24 mg/100 g), and epigallocatechin (156 ± 12 mg/100 g). Overall, this study revealed an efficient technique by which to isolate and stabilize bioactive compounds from boldo leaves, with the microcapsules being promising candidates as high added-value ingredients.

## 1. Introduction

Boldo (*Peumus boldus* M.) is a plant endemic to central and southern Chile. Its leaves have been recognized as a medicinal herb in several pharmacopoeias worldwide [1]. It has beneficial properties, such as antioxidant, anti-inflammatory, sedative, and antimicrobial properties, among others [1,2]. It has been shown that there is a high concentration of phenolic compounds in the aqueous extracts of boldo leaves, with catechins being the main component, in addition to the alkaloid boldine. Catechins have been proven to be the main free radical neutralizing component in aqueous extracts of boldo, which shows that their antioxidant activity is mainly due to catechins and flavonoids [3].

Phenolic compounds have antioxidant activity, as they can neutralize free radicals or reactive oxygen species, but these are substances highly susceptible to degradation because of external factors, such as the presence of moisture, oxygen, high temperatures, or high pH values [4]. It is for this reason that phenolic compounds must be protected in order to maintain their antioxidant capacity.

Microencapsulation is a technique used to stabilize bioactive compounds that consists of protecting a core material with the use of polymeric materials that provide protection against external factors [5]. Microencapsulation can be carried out by different processes, such as cold-drying, coacervation, and spray-drying; spray-drying is one of the most widely used techniques in the food industry because it has certain advantages over other methods [6].

Spray-drying, a unit process that converts a liquid dispersion into dry particles (<40 µm), is one of the most common methods employed for encapsulation; around 90% of encapsulated products are carried out using this process. Its main advantages are its simplicity, user-friendliness, speed, continuous operation, and cost-effectiveness. Nevertheless, this process mainly results in microcapsules that have a matrix or polynuclear structure, but the shape and morphology depend on several factors such as the wall material, the ratio of wall material to core material, and operating conditions, such as the air temperature and feed rate [7].

Therefore, the wall material used in spray-drying is important because it needs to keep the encapsulated particles stable and extend their shelf life while also being affordable and efficient. Among the main materials chosen as encapsulating matrices are polysaccharides, proteins, and lipids. Among lipids, waxes are the most commonly used for encapsulation because of their high hydrophobicity and resistance to hydrolytic degradation. Proteins are also valued for their gel-forming and foaming properties, giving rise to rigid structures for encapsulation. However, in commercial uses, polysaccharides are mainly used as encapsulants because of their properties such as viscosity and solubility. Some common polysaccharides used in spray-drying include starch, maltodextrin, chitosan, dextran, carrageenan, and gums, due to their low cost. Maltodextrin, a polysaccharide derived from the hydrolysis of starch, has different characteristics and properties compared to starch, being employed as an additive in food products and beverages and as a fat replacer due to its ability to form gels, as well as its hygroscopicity, solubility, and viscosity [7].

Microencapsulation can provide protection to phenolic compounds; however, it is important to evaluate the microencapsulation efficiency and yield (%). Both microencapsulation yield and efficiency can be affected by several factors, including the conditions of the spray-drying process (feed flow, air inlet temperature, and outlet temperature) and the ratio of encapsulant and core material used in the dissolution formulation [8].

Microencapsulation by spray-drying is an efficient technique for stabilizing bioactive phenolic compounds extracted from *P. boldus* M. leaves. Optimal extraction and spray-drying microencapsulation conditions are expected to maximize the yield, efficiency, and stability of phenolic compounds extracted from *P. boldus* M. leaves, preserving their antioxidant activity and enabling their potential application as high-value functional ingredients.

Therefore, this research aimed to develop microcapsules of boldo leaf extract by spray-drying. The extraction conditions were optimized by applying an experimental design using a response surface method to maximize the yield of phenolic compounds. The optimal extracts were considered for the spray-dryer. The yield and microencapsulation efficiency were evaluated to establish the appropriate conditions for obtaining an extract rich in phenolic compounds.

## 2. Materials and Methods

### 2.1. Plant Material

**Figure 1:**
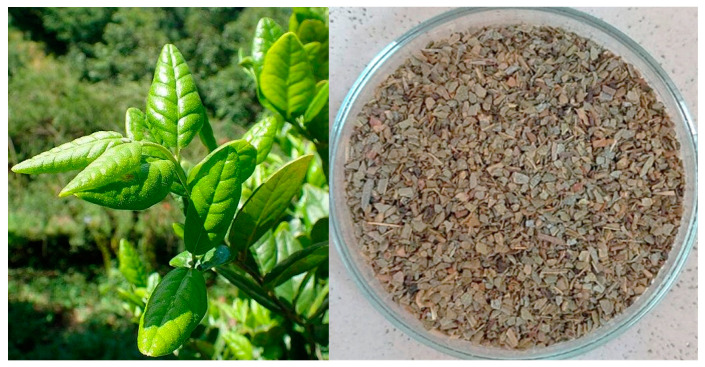
*P. boldus* M. plant and its dried leaves.

Dried boldo (*P. boldus* M.) leaves (infusion size) (Figure 1) were used. The plant material was purchased from Australis Herbolaria, with the plantations located in San Martin N° 313, Rengo, Chile.

### 2.2. Experimental Design for Phenolic Extraction

The response surface methodology (RSM) was used to optimize the extraction process of phenolic compounds and predict the responses that are affected by experimental variables. Using Statgraphics Centurion XVIII (Statistical Graphics Corp, Herdon, VA, USA), a Box-Benhken design with three independent variables was applied to evaluate the effect of temperature (°C), time (min), and solid-liquid ratio (g/mL) on the phenolic compound content of boldo leaves (response variable). The levels chosen for each independent variable are summarized in Table 1.

#### 2.2.1. Extraction Procedure

The extraction of phenolic compounds from boldo leaves was performed using the infusion extraction technique, which corresponds to a solid-liquid extraction, using different conditions in terms of temperature, time, and solid-liquid ratio, as presented in Table 2. For the extraction, 0.5 g of boldo leaf was added with distilled water in the amounts corresponding to each treatment, according to the experimental design, at 70 rpm at the temperatures indicated by the experimental design in a thermoregulated bath (model LWB-122D, LABTECH, Namyangu, Republic of Korea). They were cooled in a bath at 5 °C and filtered (Whatman 4 mm paper). The filtrate was stored and protected from light at −20 °C for further analysis.

#### 2.2.2. Total Phenolic Content

The determination of total phenolic compounds was carried out using the spectrophotometric method using a Folin-Ciocalteu assay [9]. For the determination of total phenolic compounds, 10 µL of standard solution or extract sample and 200 µL of 20% sodium carbonate were added to a microplate. Then, 50 µL of Folin-Ciocalteu reagent was added. Subsequently, samples were incubated in the dark for 30 min at room temperature. Gallic acid (1000 mg/L) was used as a reference standard (0–1000 µg/mL), and absorbance was measured at 750 nm using a UV-Vis spectrophotometer (Multiskan GO, Thermo Scientific, Vantaa, Finland). The measurements were performed in triplicate, and the results were expressed as mg GAE/g samples.

#### 2.2.3. Optimal Extraction

The results obtained for the total phenolic content of all extraction conditions were analyzed in the Statgraphics Centurion XVIII program to determine the optimal conditions. Significant differences between the values obtained were determined by multifactorial ANOVA analysis of variance with a significance level of α = 0.05.

Then, extraction was performed using these optimal conditions obtained using the response surface methodology, using 10 g of boldo leaves of infusion size (leaves of particle size range between 2 mm and 3 mm) and 1000 mL of distilled water. Subsequently, the flasks were taken to a thermoregulated bath model at 100 °C for 60 min. Then, the flasks were cooled in a water bath at a temperature of 5 °C for 10 min and filtered (Whatman 4 mm paper). Finally, the solution was concentrated in a rotary evaporator (model VV20, Heidolp, Schwabach, Germany) until the solvent was completely removed, using a temperature of 50 °C and pressure of 122 mbar to remove the water and to obtain a concentrated extract.

### 2.3. Experimental Design for Microencapsulation

These concentrated extracts were loaded into the spray-dryer. A 2^2^ factorial experimental design was applied to carry out the microencapsulation process, considering the air inlet temperature (130–150 °C) and feed flow (2–4 mL/min) as independent variables. The response variables considered for this experimental design were the encapsulation efficiency and encapsulation yield (%).

#### 2.3.1. Microencapsulation Process

For the microencapsulation by spray-drying, 1 g of boldo leaf extract, 100 mL of distilled water, and 20 g of maltodextrin were used. Then, the mixture was homogenized in a magnetic stirrer at 550 rpm. Subsequently, the mixture was loaded into a mini spray-dryer B-290 (BUCHI, Labortechnik AG, Flawil, Switzerland) while considering the process conditions of the air inlet temperature and feed flow rate indicated in Table 3, with the samples under stirring during the operation period. The suction conditions and atomization airflow rate were fixed at 536 L/h (equivalent to a 45 mm spinner height), with suction at 80% (corresponding to a drying airflow rate of 32.5 m^3^/h).

#### 2.3.2. Encapsulation Efficiency and Encapsulation Yield

In order to evaluate the encapsulation efficiency (*EE*), the content of total phenolic compounds in the microcapsules (*TPC_total_*) and the content of surface phenolic compounds in the microcapsules (*TPC_surf_*) were quantified according to Navarro-Flores et al. [10], with slight modifications, and calculated using Equation (1).

(1) $$EE\;\left(\%\right)=(\frac{{TPC}_{total}-{TPC}_{surf}}{{TPC}_{total}})\times 100$$

To determine the content of total phenolic compounds in the microcapsules (*TPC_total_*), aliquots of 200 mg of microcapsules were taken, and 2 mL of a mixture of ethanol, acetic acid, and distilled water (50:8:42 *v*/*v*/*v*) was added and mixed in a vortex for 1 min. The samples were then sonicated in an ultrasonic bath (Ney 300 ultrasonic, Yucaipa, California, CA, USA) at 25 °C for 20 min and centrifuged (model Z325K, HERMLE Labortechnik, Wehingen, Germany) for 5 min at 11,200× *g*.

To determine the content of surface phenolic compounds in the microcapsules (*TPCsurf*), 200 mg of microcapsules were used, and 2 mL of ethanol:methanol (1:1 *v*/*v*) were added and vortexed for 1 min. They were then centrifuged for 5 min at 11,200× *g*. The supernatant was filtered through a 0.45 µm filter.

The *TPC_total_* and *TPCsurf* were determined using the Folin-Ciocalteu method [8]. For the total phenolic content, 10 µL of the sample was mixed with 200 µL of 20% calcium carbonate for 2 min. Then, 50 µL of Folin-Ciocalteu reagent (diluted 1/5) was added. The mixture was then incubated for 30 min under dark conditions at room temperature, and the absorbance at 750 nm was measured.

The microencapsulation yield was determined by considering the relationship between the mass of the microcapsules obtained after the spray-drying process and the expected mass of the microcapsules according to the number of solids entering the equipment [11]. The encapsulation yield was calculated using Equation (2):

(2) $$Yield\;(\%)=\left(\frac{MM}{CMM+WMM}\right)\times 100$$

where *MM* is the microcapsule mass obtained after spray-drying (g); *CMM* is the core material mass (boldo leaf extract) (g); and *WMM* is the wall material mass (maltodextrin) (g).

#### 2.3.3. Water Activity and Moisture of Microcapsules

The moisture of microcapsules was determined using an infrared moisture analyzer (model LP16, Mettler-Toledo, Greyfensee, Switzerland), with a temperature range between 50–160 °C and operating time settings. The temperature used was 120 °C until the constant weight of the sample. The results were expressed as % moisture.

Water activity was determined using a water activity analyzer (model CX3T, Aqualab, Washington, WA, USA) using 1 g of microcapsules.

#### 2.3.4. Scanning Electron Microscopy and Zeta Potential of Microcapsules

The morphology of the microcapsules was investigated by scanning electron microscopy (SEM). The size and geometric shape of the microcapsules were analyzed using a Carl Zeiss EVO MA 10 scanning electron microscope (Carl Zeiss SMT Ltd., Cambridge, UK) equipped with an energy-dispersive X-ray spectroscopy detector (X-ACT, Oxford Instruments, Abingdon, UK), with an acceleration voltage of 10 kV and a pressure of 6 × 10^−6^ mbar. The samples were fixed on graphite tape, subjected to a thin coating of gold (Au) (Denton Vacuum Desk IV, Denton Vacuum, Moorestown, NJ, USA), and analyzed under high vacuum to obtain high-resolution SEM images. The secondary electron detector EDX was used to evaluate the morphology and topography of the samples. In addition, zeta potential was measured by light scattering at 25 °C on a Zetasizer (Nano ZS90, Malvern Panalytical Ltd., Malvern, UK). For this purpose, particles were suspended in Milli-Q water and gently shaken to disperse without dissolving. The results were expressed in mV.

#### 2.3.5. Fourier Transform Infrared Spectroscopy (FTIR)

FTIR spectra of microcapsules, maltodextrin, and boldo extract were studied using a Perkin-Elmer Spectrum UATR Two spectrometer (Perkin Elmer Inc., Shelton, CT, USA) in mid-infrared mode, equipped with a universal ATR (Attenuated Total Reflectance). Scanning conditions were as follows: transmission mode in the range of 4000–500 cm^−1^, with a scan rate of 0.20 cm s^−1^ and four accumulations with a resolution of 4 cm^−1^. Spectra were acquired and processed using Spectrum software (version 10.5.1.581).

### 2.4. Bioactive Characterization of Microcapsules

#### 2.4.1. DPPH Free Radical Scavenging Method

The extracts obtained for total phenolic compounds were used to determine the antioxidant activity of microcapsules. The activity of free radical scavenging was determined using the 2,2-diphenyl-1-picrylhydrazyl (DPPH) method, following the methodology performed by Moreira et al. [12]. For the assays, 100 µL of extracts were used, and 100 µL of DPPH solution was added. They reacted for 30 min in the dark at room temperature, and then the absorbance at 517 nm was measured using a UV-Vis spectrophotometer (Multiskan GO, Thermo Scientific, Vantaa, Finland). Trolox was used as a reference standard at different concentrations (0–100 µM). The results were expressed as µM of Trolox equivalents (TE) per g of microcapsules.

#### 2.4.2. ABTS Free Radical Scavenging Method

The ABTS [2,20-azino-bis(3-ethylbenzothiazoline-6-sulphonic acid)] cation radical method was performed using the methodology developed by Re et al. [13]. For the assays, 100 µL of extracts were used, and 100 µL of ABTS solution was added. They reacted for 7 min in the dark at room temperature, and then the absorbance at 734 nm was measured using a UV-Vis spectrophotometer (Multiskan GO, Thermo Scientific, Vantaa, Finland). Trolox (10 mM) was used as a reference standard in different concentrations (0.20–3.00 mmol/L). Trolox was used as a reference standard at different concentrations (0–100 µM). The results were expressed as µM of TE per g of microcapsules.

#### 2.4.3. FRAP Method

The Ferric Reducing Antioxidant Power (FRAP) method was adopted from Benzie and Strain [14]. Briefly, the FRAP reagent was prepared fresh daily by mixing 25 mL of acetate buffer (pH 3.6), 2.5 mL of TPTZ solution (10 mM) prepared in 40 mM HCl, and 2.5 mL of FeCl_3_ 6H_2_O solution (20 mM) at a volume ratio of 10:1:1, respectively. The FRAP solution was heated at 37 °C. For the assays, 150 µL of extracts were used, and 285 µL of FRAP solution was added. They reacted for 30 min in the dark at 37 °C, and then the absorbance at 539 nm was measured using a UV-Vis spectrophotometer (Multiskan GO, Thermo Scientific, Vantaa, Finland). Trolox was used as a reference standard at different concentrations (0–800 µM). The results were expressed as µM of TE per g of microcapsules.

#### 2.4.4. Identification and Quantification of Phenolic Compounds

For the analysis, first, a release of the phenolic compounds in the microcapsules was performed according to the methodology explained by Navarro-Flores et al. [10], and the pure extract was also analyzed.

Identification of the phenolic compounds was performed with an Agilent HPLC 1200 series model (Agilent Technologies, Santa Clara, CA, USA) equipped with a diode array detector (DAD) (model G1316A), a high-pressure quaternary pump (model G1311A), a degasser (model G1322A), and an autosampler (model G1329A) controlled by the software “Agilent ChemStation (V04.02.096)”, based on the methodology described by Quispe-Fuentes et al. [15]. A Kromasil 100–5 C18 column (25.0 cm × 0.46 cm; 5 μm particle size waters; Eka Chemicals, Bohus, Sweden) was used. Mobile phase, pumped at a flow rate of 0.7 mL/min, consisted of 0.1% aqueous formic acid (solvent A) and acetonitrile (solvent B), and the elution gradient was initially set at: 87% A and 13% B from time 0 to 16 min; 48% A and 52% B from time 16 to 23 min; 60% A and 40% B from time 23 to 25 min; 87% A and 13% B from time 25 to 30 min. The injection volume was 10 μL. Chromatograms were recorded at 280, 310, and 370 nm at 25 °C, and compounds in each sample were tentatively identified based on their elution order retention times and spectrum features as compared to authentic standards analyzed under the same conditions. Identified compounds were finally quantified using calibration curves of the standard compounds. The results were expressed in mg of phenolic compound per 100 g of microcapsules.

### 2.5. Statistical Analysis

The results were expressed as the mean ± standard deviation. The mean comparisons were carried out using an analysis of variance (ANOVA) using Statgraphics Centurion XVIII (Statistical Graphics Corp, Herdon, VA, USA). The multiple range test (MRT) was also included in the statistical program to demonstrate the existence of homogeneous groups within each quantified parameter, which correspond to microencapsulation efficiency and yield, total phenolic compound content, identified phenolic compounds, antioxidant capacity, water activity, and moisture. The significant differences were established as (*p* < 0.05). All determinations were carried out in triplicate.

## 3. Results and Discussion

### 3.1. Optimal Phenolic Extraction

A design of experiments (DOE) was planned to obtain phenolic compounds from boldo leaves. To optimize the extraction procedure, the effect of process variables on the TPC was evaluated on a Box-Behnken design with three independent variables: temperature, time, and solid-liquid ratio time. The results of the interaction effects of the three independent variables studied on phenolic compound extraction are shown in Table 2.

The content of phenolic compounds in the extractions carried out ranged between 2.05 and 37.78 mg GAE/g, with the maximum values higher than those obtained in previous research using pretreated wild *P. boldus* M. leaves, where values of 34.30 mg GAE/g were reported [16]. However, Torres-Vega et al. [17] reported a total phenolic content in extracts from boldo leaves of about 70 mg GAE/g, extracted by heat and stirring using water as a solvent. These differences in TPC values may be due to the extraction conditions used, such as temperature, particle size, solid-liquid ratio, and time [18].

**Figure 2:**
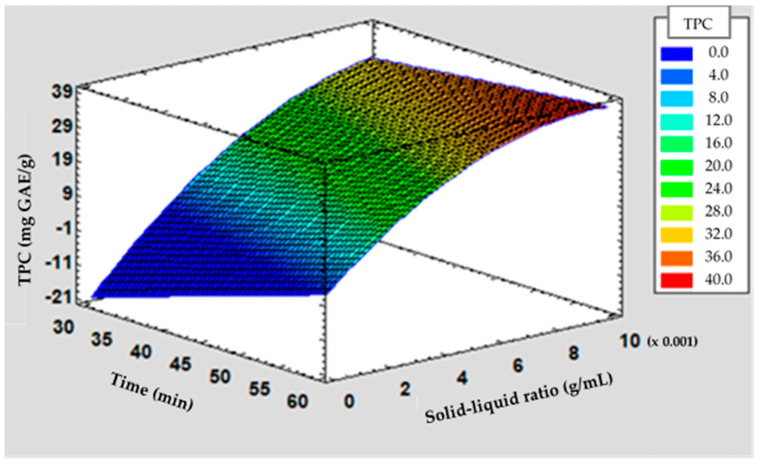
Response surface plot representing the effects of time and solid-liquid ratio and temperature on Total Phenolic Content (TPC) from boldo leaves, with the temperature constant at 100 °C. Lower values are represented in blue and higher values in red.

As can be seen in the response surface plot (Figure 2), the correlation between the factors of solid-liquid ratio and time in stable temperature at 100 °C can be exploited to increase the affinity and specificity of the extraction. The TPC values were higher, increasing the time (60 min) and the solid-liquid ratio. Nevertheless, the values of TPC were lower, using extraction factors for a shorter time (30 min) and a lower solid-liquid ratio (blue-green surface).

The higher yield of phenolic compounds can be attributed to the combined effect of high temperature (100 °C), prolonged extraction time (60 min), and a solid-liquid ratio of 1:100, which enhanced the migration and solubilization of bioactive compounds by increasing the contact between the plant matrix and the solvent. Consequently, these factors contributed to a higher yield of phenolic compounds, particularly those that are polar and tolerant to high temperatures [19]. Hence, TPC values indicated that increasing the extraction time while increasing the solid-liquid ratio and keeping the temperature constant at 100 °C increases the extraction yield of these bioactive compounds (red surface).

The statistical analysis of the 15 runs revealed that the TPC is best described by a second-order linear regression model (Equation (3)), with the independent variables: temperature (A), time (B), and solid-liquid ratio (C):

TPC = 74.9608 − 1.197 × A − 1.348 × B + 5740.8 × C + 0.021 × A × B + 59.5 × A × C − 49.875 × B × C − 480926 × C^2^ (3)

In the proposed model, the regression coefficient R^2^ was 0.8651, indicating accuracy and a good fit of the experimental data with the calculated data, and the adjusted coefficient R^2^ was 0.7303, which verifies the adequacy of the model, as in order to make a prediction, an adjusted coefficient of determination of at least 70% is recommended [20].

According to the results, the extraction process conditions in which a maximum content of phenolic compounds from boldo leaves can be obtained were a temperature of 100 °C, a time of 60 min, and a solid-liquid ratio of 1:100 (g/mL). The extraction was carried out based on the conditions granted by the experimental design, obtaining a content of total phenolic compounds of 39.7 ± 2.13 mg GAE/g, which, when compared to the optimal value predicted by the optimization, has an error of 2.2%, because, from the regression model, it was determined that the maximum content of phenolic compounds that can be obtained in the aqueous extract of boldo leaves under the optimal conditions is 38.84 mg GAE/g. This error is justifiable, as in the linear regression models used to study the response variable, it was only able to explain 73.03% of the variability of the results; for this reason, the linear regression model may underestimate or overestimate the predicted value.

The optimal extract with a high TPC content was microencapsulated by spray-drying, and different encapsulation conditions were applied to improve its stability and study the potential of boldo leaves for further applications.

### 3.2. Characterization of Microcapsules with the Optimal Boldo Extract

The determination of microencapsulation yield and efficiency are the most important criteria by which to assess whether a correct encapsulation of the extract was performed. These parameters can be influenced by several factors, such as the air inlet temperature, the feed flow, and the concentration of wall and core material used in the formulation of the solution to be encapsulated [8].

In the present study, a total of four microencapsulation treatments were carried out, in which the effect of the air inlet temperature and the feed flow were determined; it should be noted that both factors are conditions of the spray-drying process. Table 3 shows the results obtained for microencapsulation yield and efficiency (*EE*).

The yields obtained ranged between 71.76 and 79.31%, and two homogeneous groups were presented, which means that there is a statistically significant difference (*p* < 0.05) between the treatments carried out with a feed flow of 2 and 4 mL/min. It was determined that the treatments that were performed with a feeding flow of 2 mL/min had higher encapsulation yields than the treatments carried out with a feeding flow of 4 mL/min. Therefore, with a lower feeding flow, a higher encapsulation yield was obtained. This result could be because the sample enters the equipment more slowly, achieving greater evaporation of the water present in the atomized solution; consequently, the microcapsules obtained have lower humidity, achieving less adherence to the equipment walls and thus increasing the yield [21]. According to previous research, a microencapsulation of green tea extract using maltodextrin and gum rabic as wall material reported yields ranging between 71.41 and 88.04% [4], showing similar yield values to those obtained in the present study. In addition, in the microencapsulation of an extract of phenolic compounds from chipilin leaves using maltodextrin, gum Arabic, and soy proteins as wall material, microencapsulation yields between 46 and 64% were obtained [10], which were lower than those reported in this research.

Regarding the microencapsulation efficiency, which relates to the content of total phenolic compounds in the microcapsules and those found on their surface, values between 90.58 and 97.31% were obtained. There is a significant difference (*p* < 0.05) in the *EE* in those treatments carried out at lower temperatures (130 °C) compared to the ones carried out at higher temperatures (150 °C). In the microcapsules at 150 °C, a lower *EE* was obtained, showing that a higher air temperature could generate a deterioration of the phenolic compounds, resulting in a lower *EE* [22]. In previous studies where a microencapsulation of phenolic compound extract from chipilin leaves was reported using maltodextrin as a wall material, an *EE* between 63% and 92% was obtained, lower than those obtained in the present study [10].

The water activity and moisture (%” of ’ll obtained microcapsules are presented in Table 3. It can be seen that in the water activity of the microcapsules, there are no significant differences between the treatments. In the case of moisture, there is a significant difference (*p* < 0.05) between the treatment carried out at lower temperatures and the treatments at higher temperatures, where a lower moisture was obtained in those treatments with a high temperature and higher moisture than in treatments using a lower temperature.

Treatment 2 showed the lowest water activity, which correlates to the lowest moisture content of the samples. On the contrary, Treatment 1 had the highest water activity, which correlates to the highest moisture value. It is worth explaining that the treatment with the highest moisture content was the sample that was obtained by applying a high temperature and a low feed flow, which allowed the droplets to be atomized later in the spray-drying process, causing greater evaporation and, consequently, a powder with low moisture content. Regarding the air inlet temperature, the higher it is, the higher the heat transfer rate to the particles, which provides a greater driving force for moisture evaporation [23].

In this study, microcapsules with a moisture content between 2.10 and 3.29% were obtained, which are similar to those reported previously in microcapsules of chipilin phenolic compounds extract that contained a moisture content between 3 and 4% [10] and in the microencapsulation study of green tea phenolic-rich extract carried out by Tengse et al. [24], in which moisture contents ranged between 2.1 and 3.1%. It is important to consider that a moisture percentage of less than 5% is necessary for good stability and effective storage of the microcapsules.

Regarding the water activity of the microcapsules, values varied between 0.24 and 0.29. Similar values were obtained in previous studies in which water activity values between 0.25 and 0.39 were reported [10]. It has been shown that a water activity of less than 0.3 is favorable for the stability of the powder, since there is less free water available for the growth of microorganisms and biochemical reactions, therefore, its shelf life is increased [25].

**Figure 3:**
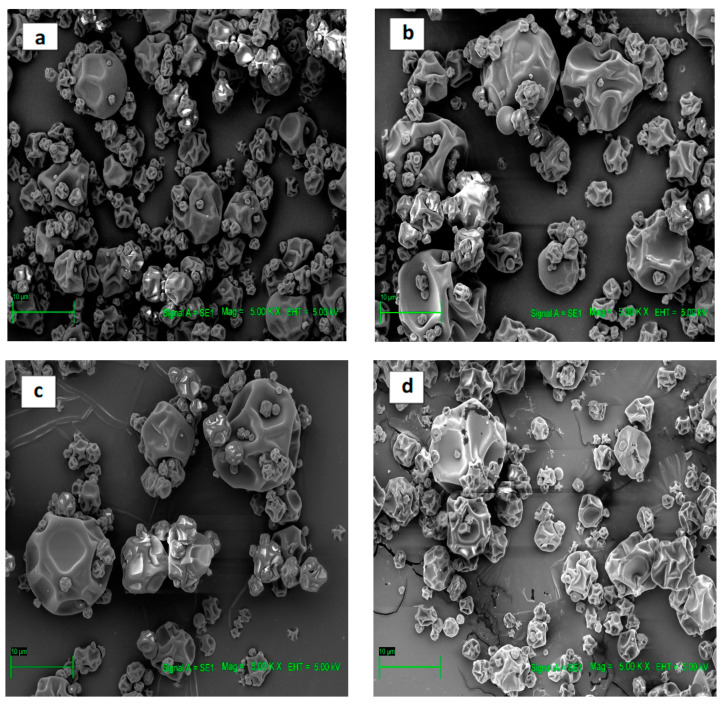
SEM micrographs of microcapsules. (**a**) Treatment N°1 microencapsulation (130 °C, 2 mL/min); (**b**) Treatment N°2 microencapsulation (150 °C, 2 mL/min); (**c**) Treatment N°3 microencapsulation (130 °C, 4 mL/min); (**d**) Treatment N°4 microencapsulation (150 °C, 4 mL/min).

Based on the scanned electron microscopic images (Figure 3), spherical maltodextrin particles of different sizes can be detected, which in some cases presented indentations on their external part, which were produced with the increase in the evaporation temperature during spray-drying. These findings are similar to previous research. They reported that powders produced with maltodextrin carriers showed relatively uniform, smooth, spherical particles with large agglomerates, since wall materials affect the surface structure and morphology of spray-dried particles [18,26,27].

Regarding the results of the zeta potential, microencapsulates with different treatments reported negative values between −11.7 and −20.1 mV (Table 3). The data show that as the feed flow increases, the negative zeta potential of the product also increases (*p* < 0.05). In a solution of particles with high zeta potential, the particles will repel each other. As a consequence, the solution will be well dispersed.

**Figure 4:**
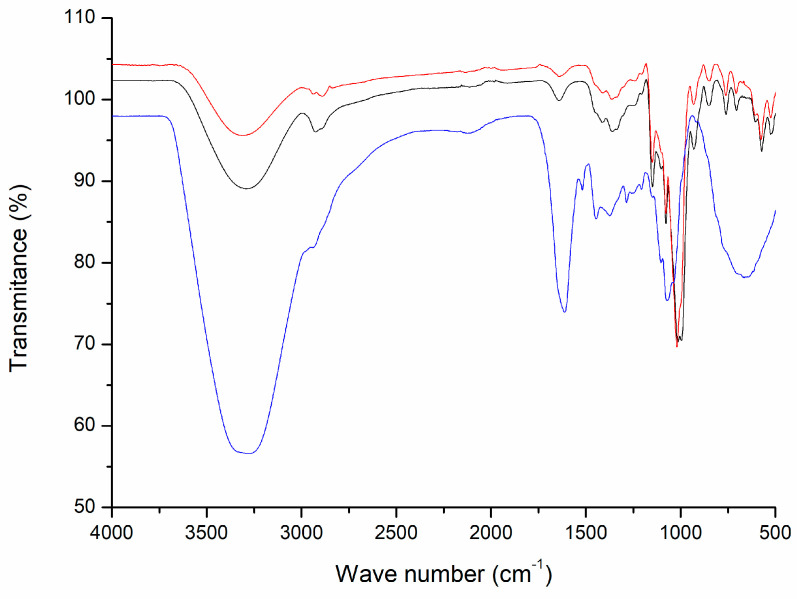
FTIR spectra for different samples. BOLDO extract (blue line), maltodextrin (black line), and Treatment 3 as a model (red line).

Figure 4 shows the FTIR spectra for the polyphenol extract, maltodextrin, and encapsulated extract (Treatment 3 was used as a model, considering that all spectra exhibit similar FTIR spectra (Figure S1)). The broad vibrational band at 3313 cm^−1^ is attributed to O–H stretching vibrations due to hydroxyl groups on maltodextrin and polyphenol compounds. The bands at 2924 and 2871 cm^−1^ are associated with the asymmetric and symmetric C–H stretching vibrations of alkanes [28]. The intense peak at 1614 cm^−1^ can be assigned to C=O stretching vibrations of carbonyl groups, a characteristic functional group on the polyphenol structure. On the other hand, maltodextrin and encapsulated extract exhibit a band around 1640 cm^−1^, which can be attributed to water absorption. The region between 1250 and 1000 cm^−1^ is attributed to hydroxyl vibration due to C–O stretching and C–O–H bending. The polyphenol extract FTIR shows vibration for phenol (at 1205 cm^−1^), tertiary alcohol (1148 cm^−1^), and primary alcohol (1080 cm^−1^); all of them can be found in the polyphenol extract. Oppositely, the maltodextrin spectrum and those obtained for the encapsulation with Treatment 3 only show a vibration band at 1017 cm^−1^, which is related to the primary hydroxyl content on maltodextrin [28].

### 3.3. Content of Phenolic Compounds in Microcapsules: Phenolic Profile and Antioxidant Properties

As reported in Table 4, five phenolic compounds were identified and quantified (pyrogallol, catechin, epicatechin, epigallocatechin, and rutin) in the concentrated boldo leaf extract obtained with the optimal extraction conditions. Regarding the flavonol fraction identified, it was composed of the flavan-3-ols catechin, epicatechin, and epigallocatechinn, the flavonoid glycoside rutin, and pyrogallol. Catechin resulted in the most abundant phenolic compound (445 ± 37 mg/100 g), followed by pyrogallol (304 ± 24 mg/100 g) and epigallocatechin (156 ± 12 mg/100 g). Epicatechin and rutin were also present but in lower concentrations (99–143 mg/100 g).

These results are similar to other studies that reported the content of catechin and epicatechin in commercial infusions of boldo leaves [18]. Furthermore, Hirschmann et al. [3] reported that catechin compound was the main phenolic compound identified in aqueous extracts of boldo and was also considered the main component that provides antioxidant capacity.

As reported in Table 4, these phenolic compounds were also identified and quantified in the microcapsules with boldo leaf extracts obtained under different conditions. Catechin was the major one found (56–62 mg/100 g), followed by pyrogallol (25–37 mg/100 g) and epigallocatechin (≈20 mg/100 g). Nevertheless, statistically significant differences were obtained in the pyrogallol and catechin content between the microencapsulation samples. The treatment was carried out with a faster feed flow of 4 mL/min and a higher temperature (150 °C) in the spray-drying process, and a high concentration of these bioactive compounds was obtained. Therefore, the conditions used in the microencapsulation process influence the concentration of these phenolic compounds. These results may be related to the fact that by entering the solution faster into the spray-drying process, a lower degradation of these compounds is allowed and, consequently, a higher quantification of phenolic compounds is achieved.

**Table 4:** Phenolic compounds identified and quantified in the boldo extract and the microcapsules containing the boldo extract by HPLC ^1^.

Identified Compounds		Encapsulated Powders			
	Boldo Extract	130 °C, 2 mL/min	150 °C, 2 mL/min	130 °C, 4 mL/min	150 °C, 4 mL/min
	mg/100 g Extract	mg/100 g of Microcapsules			
1. Pyrogallol	303.8 ± 23.97	25.49 ± 0.25 ^d^	29.88 ± 0.50 ^c^	32.64 ± 0.50 ^b^	37.40 ± 0.74 ^a^
2. Catechin	445.3 ± 37.39	56.38 ± 0.24 ^d^	59.31 ± 0.24 ^c^	60.79 ± 0.19 ^b^	62.22 ± 0.49 ^a^
3. Epicatechin	143.3 ± 14.95	15.55 ± 0.22 ^c^	18.51 ± 1.00 ^b^	16.95 ± 0.22 ^a^	16.39 ± 0.11 ^ac^
4. Epigallocatechin	155.9 ± 12.11	20.53 ± 0.36 ^ab^	20.65 ± 0.07 ^ab^	20.79 ± 0.11 ^a^	20.39 ± 0.06 ^b^
5. Rutin	99.18 ± 11.55	13.45 ± 0.15 ^b^	13.43 ± 0.09 ^b^	14.19 ± 0.17 ^a^	13.40 ± 0.08 ^b^

^1^ Means ± standard deviation in the same row followed by different lowercase letters indicate statistically significant differences at *p* ≤ 0.05 for each sample (*n* = 3).

In the case of the epigallotechin and rutin compounds, two homogeneous groups were identified, where a higher content of these compounds was obtained in the treatment with a lower temperature (130 °C) and a higher feed flow (4 mL/min). Thus, the concentration of these bioactive compounds was influenced by the temperature and feed flow used during the encapsulation process. These results may be related to the fact that, when using lower temperatures, there is a lower loss of these components during the spray-drying process because they are less susceptible to degradation compared to the use of higher temperatures [24]. In addition, a faster feed flow allows for less degradation of the compounds, as they undergo the spray-drying process for less time.

The results of the total phenolic content of the microcapsules are shown in Table 5. As can be seen, the microcapsules carried out at a temperature of 150 °C had a lower content of total phenolic compounds (5.38–5.49 mg GAE/g) compared to the microcapsules obtained at a temperature of 130 °C (5.75–5.95 mg GAE/g), showing statistically significant differences between them. This fact could be due to the degradation of the phenolic compounds susceptible to high temperatures [24].

The DPPH, ABTS, and FRAP assay results revealed the different antioxidant activities of microcapsules containing boldo leaf extracts (Table 5). DPPH and ABTS tests evaluate the in vitro antiradical activity, and FRAP assay the reducing potential of the microcapsules.

There is no statistically significant difference in the DPPH antioxidant capacity of microcapsules obtained under different conditions (≈185–187 µM TE/g). However, for the microcapsules carried out at lower temperatures and feed flows (2 mL/min), significantly higher FRAP and ABTS antioxidant capacities were found (2317 ± 3 and 100 ± 4 µM TE/g, respectively). Furthermore, there was a directly proportional relationship between the content of total phenolic compounds and the antioxidant capacity of the microcapsules, since the treatments carried out at a higher temperature have a lower content of total phenolic compounds and antioxidant capacity; on the contrary, the treatments carried out at a lower temperature (130 °C) have a higher content of total phenolic compounds and antioxidant capacity.

**Table 5:** Total Phenolic Content (TPC) and antioxidant activity of microcapsules ^1^.

Treatment	Inlet Air Temperature (°C)	Feed Flow (mL/min)	FRAP(µM TE/g Sample)	ABTS(µM TE/g Sample)	DPPH(µM TE/g Sample)	TPC(mg GAE/g Sample)
1	130	2	2316.80 ± 2.92 ^a^	99.86 ± 3.56 ^a^	186.53 ± 1.97 ^a^	5.75 ± 0.14 ^ab^
2	150	2	2016.48 ± 38.74 ^b^	87.99 ± 2.12 ^ab^	185.72 ± 0.20 ^a^	5.43 ± 0.26 ^b^
3	130	4	2017.00 ± 0.00 ^b^	83.03 ± 2.28 ^b^	187.12 ± 0.31 ^a^	5.95 ± 0.28 ^a^
4	150	4	2086.26 ± 39.47 ^b^	84.76 ± 9.29 ^b^	186.46 ± 0.62 ^a^	5.38 ± 0.11 ^b^

^1^ Means ± standard deviation in the same column followed by different lowercase letters indicate statistically significant differences at *p* ≤ 0.05 for each sample (*n* = 3).

These results are similar to other studies on the microencapsulation of phenolic compounds in green tea extract that reported that the higher the content of phenolic compounds obtained in the microcapsules, the higher the antioxidant capacity [24], and also to that of Navarro-Flores et al. [10] regarding the microcapsules of chipilin extract in which it was determined that the results of the antioxidant capacity were proportional to the content of phenolic compounds, which indicates that phenolic compounds have an important role in the antioxidant capacity of the microencapsulated extracts.

In this context, Vargas et al. [29], using maltodextrin as a wall material for the microcapsules of fruit pulp by spray-drying, also reported that the antioxidant activity was attributed to the content of phenolic compounds obtained. They attributed this to the oxidation of phenolic compounds observed during spray-drying, which enhanced the samples’ capture activity. They concluded that partially oxidized polyphenols can significantly enhance radical scavenging efficacy, particularly compared to polyphenols that have not undergone oxidation.

On the other hand, Cruz-Molina et al. [30] reported that the microencapsulation of green tea extracts using maltodextrin as the wall material significantly improved the thermal stability of the catechins and antioxidant compounds, showing that microencapsulation by spray-drying not only preserved the antioxidant capacity of the extract during storage but also protected the phenolic compounds from adverse conditions, including high temperatures and pH changes, and this effect was attributed to the formation of an effective physical barrier of maltodextrin that prevented the degradation of the bioactive compounds.

Therefore, these data showed boldo leaves are a valuable source of potentially interesting phytochemicals, such as flavonoids, and could constitute a workable source of antioxidant compounds.

## 4. Conclusions

This study evaluated the implementation and optimization of phenolic compounds from boldo leaves and their encapsulation by spray-drying, providing new information on an efficient process that could be scaled up to an industrial level.

The results showed, at the laboratory level, that the optimal conditions for the extraction of bioactive compounds were the employment of a temperature of 100 °C, a time of 60 min, and a solid-liquid ratio of 1:100. Moreover, it was reported that the air inlet temperature and the spray-drying feed flow influence the yield and the microencapsulation efficiency. The best conditions for high microencapsulation efficiency (97%) were an air inlet temperature of 130 °C and a feed flow of 4 mL/min, obtaining a good encapsulation yield (71%) and a high content of phenolic compounds and antioxidant capacity.

According to the findings of this research, the boldo leaf microcapsules had a good amount of bioactive compounds, especially flavonoids such as catechin, which are representative of promising candidates for various applications such as food or cosmetics, among others. Nevertheless, further research about the phenolic content over time needs to be carried out in order to determine the stability of microcapsules, and the incorporation of the microcapsules in some food products could be evaluated to improve the nutritional values of the food formulations in terms of antioxidant properties.

## Figures and Tables

**Table 1:** Coded variable levels of the experimental design.

Independent Variable	Units	Code Levels		
		−1	0	+1
Temperature	°C	80	90	100
Time	min	30	45	60
Solid-liquid ratio	g/mL	1:100	1:166	1:500

**Table 2:** The BBD matrix of the experimental design and results of Total Phenolic Content (TPC) ^1^.

Experiment	Independent Variables			Response Variable
	Temperature (°C)	Time (min)	Solid-Liquid Ratio (g/mL)	TPC (GAE mg/g Sample)
1	90 (0)	45 (0)	1:166 (0)	23.04 ± 0.03
2	80 (−1)	45 (0)	1:100 (+1)	28.86 ± 0.06
3	90 (0)	60 (+1)	1:100 (+1)	32.26 ± 0.11
4	100 (+1)	60 (+1)	1:166 (0)	28.93 ± 0.04
5	90 (0)	45 (0)	1:166 (0)	34.13 ± 0.05
6	80 (−1)	60 (+1)	1:166 (0)	22.89 ± 0.02
7	90 (0)	60 (+1)	1:500 (−1)	19.94 ± 0.06
8	100 (+1)	45 (0)	1:100 (+1)	37.78 ± 0.01
9	100 (+1)	30 (−1)	1:166 (0)	20.23 ± 0.02
10	90 (0)	30 (−1)	1:100 (+1)	26.95 ± 0.07
11	100 (+1)	45 (0)	1:500 (−1)	2.05 ± 0.01
12	90 (0)	30 (−1)	1:500 (−1)	2.65 ± 0.07
13	80 (−1)	30 (−1)	1:166 (0)	26.70 ± 0.02
14	90 (0)	45 (0)	1:166 (0)	31.95 ± 0.01
15	80 (−1)	45 (0)	1:500 (−1)	2.65 ± 0.02

^1^ Means ± standard deviation.

**Table 3:** Results of microcapsules characterization ^1^.

Treatment	Inlet Air Temperature (°C)	Feed Flow (mL/min)	Water Activity	Moisture (%)	Yield (%)	Efficiency (%)	Z Potential (mV)
1	130	2	0.29 ± 0.04 ^a^	3.29 ± 0.17 ^a^	79.91 ± 0.84 ^a^	93.53 ± 1.10 ^ab^	−14.0 ± 3.19 ^ab^
2	150	2	0.24 ± 0.05 ^a^	2.10 ± 0.25 ^b^	76.56 ± 0.67 ^a^	90.55 ± 1.34 ^b^	−11.7 ± 3.82 ^b^
3	130	4	0.28 ± 0.03 ^a^	2.75 ± 0.36 ^ab^	71.33 ± 0.01 ^b^	97.02 ± 2.63 ^a^	−18.7 ± 3.49 ^a^
4	150	4	0.26 ± 0.04 ^a^	2.52 ± 0.11 ^b^	73.43 ± 2.37 ^b^	88.06 ± 2.36 ^b^	−20.1 ± 3.12 ^a^

^1^ Means ± standard deviation in the same column followed by different lowercase letters indicate statistically significant differences at *p* ≤ 0.05 for each sample (*n* = 3).

## Data Availability

All the data supporting this research article are contained within the paper/Supplementary Materials. Raw data will be made available by the authors upon reasonable request.

## Supplementary Materials

The following supporting information can be downloaded at: https://www.mdpi.com/article/10.3390/antiox13121568/s1, Figure S1: FTIR spectra for different samples. Comparison between several treatments: treatment 1 (pink line), treatment 2 (blue line), treatment 3 (red line) and treatment 4 (black line); Figure S2: HPLC chromatograms of the microcapsules obtained from the 4 treatments carried out. (a) Treatment 1 microencapsulation (130 °C, 2 mL/min); (b) Treatment 2 microencapsulation (150 °C, 2 mL/min); (c) Treatment 3 microencapsulation (130 °C, 4 mL/min); (d) Treatment 4 microencapsulation (150 °C, 4 mL/min). The phenolic compounds identified are: (1) pyrogallol (2) catechin (3) epicatechin (4) epigallocatechin (5) rutin.
