# Supplementary material for: Bioactive Content and Antioxidant Properties of Spray-Dried Microencapsulates of Peumus boldus M. Leaf Extracts
Source: Antioxidants (Basel). 2024 Dec 20;13(12):1568. doi: 10.3390/antiox13121568 (PMC11673136; doi:10.3390/antiox13121568)
Supplement: Supplementary file 1 [file antioxidants-13-01568-s001.zip › antioxidants-3313402-supplementary.pdf]

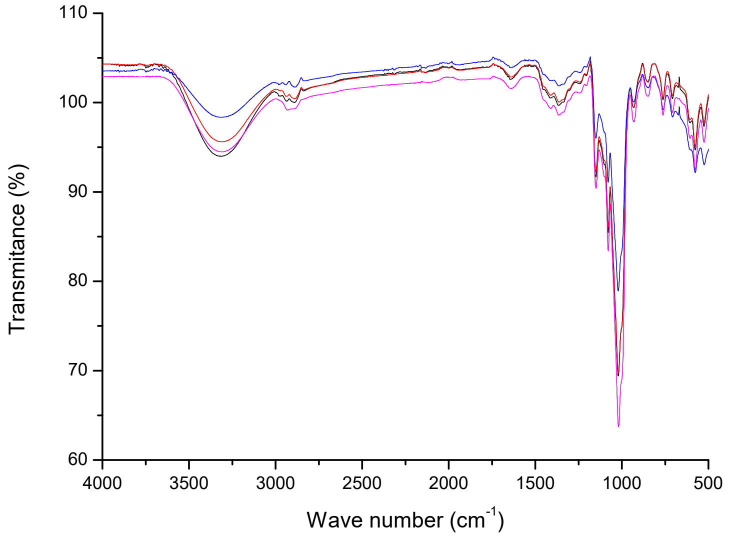

Figure S1. FTIR spectra for different samples. Comparison between several treatments: treatment 1 (pink line), treatment 2 (blue line), treatment 3 (red line) and treatment 4 (black line)

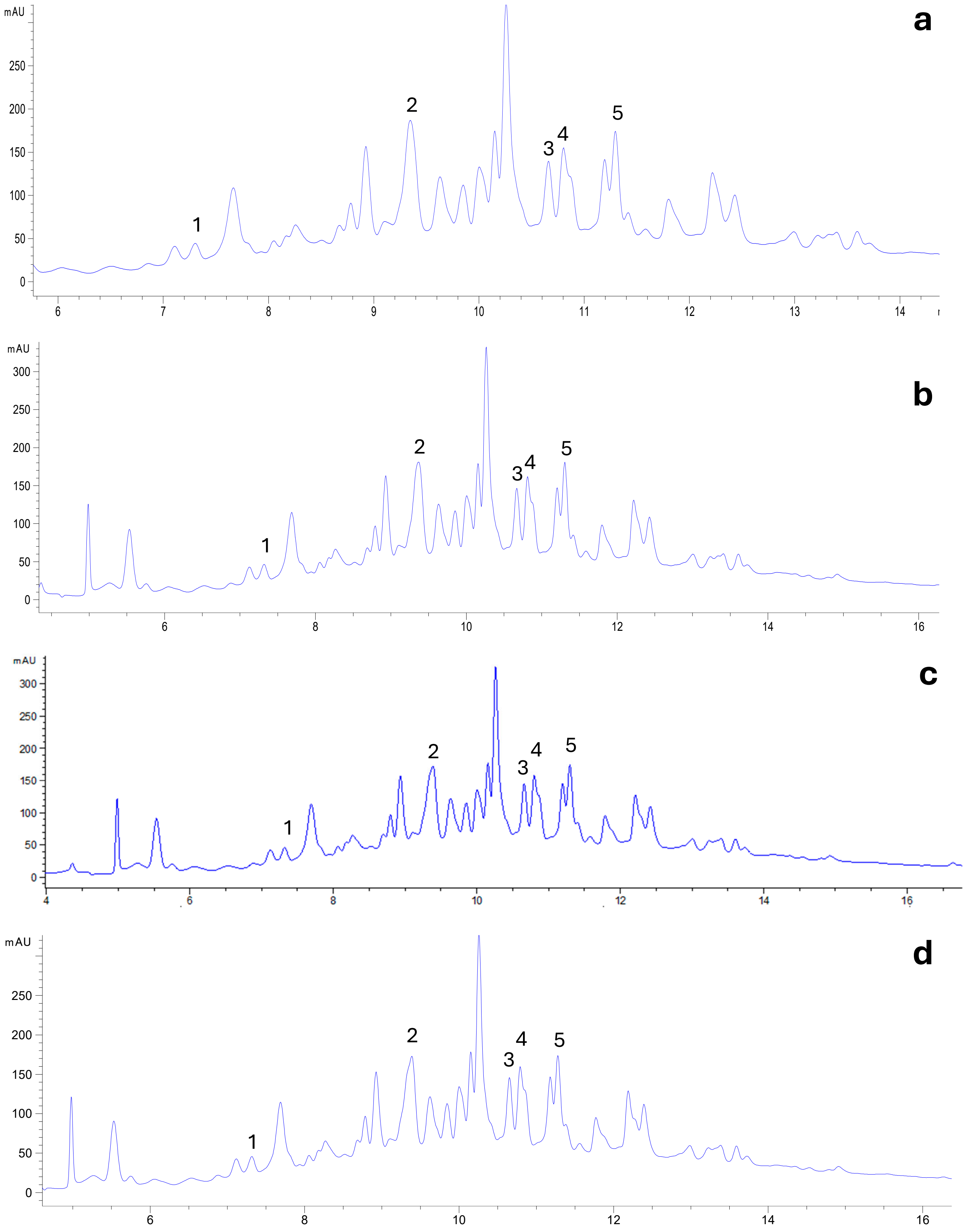

Figure S2. HPLC chromatograms of the microcapsules obtained from the 4 treatments carried out. (a) Treatment 1 microencapsulation (130°C, 2 mL/min); (b) Treatment 2 microencapsulation (150°C, 2 mL/min); (c) Treatment 3 microencapsulation (130°C, 4 mL/min); (d) Treatment 4 microencapsulation (150°C, 4

mL/min). The phenolic compounds identified are: 1) pyrogallol 2) catechin 3) epicatechin 4) epigallocatechin 5) rutin.
